# Fungal Endophyte-Mediated Crop Improvement: The Way Ahead

**DOI:** 10.3389/fpls.2020.561007

**Published:** 2020-10-27

**Authors:** Vijaya R. Chitnis, Trichur S. Suryanarayanan, Karaba N. Nataraja, S. Rajendra Prasad, Ralf Oelmüller, R. Uma Shaanker

**Affiliations:** ^1^School of Ecology and Conservation, University of Agricultural Sciences, GKVK, Bangalore, India; ^2^Vivekananda Institute of Tropical Mycology (VINSTROM), Ramakrishna Mission Vidyapith, Chennai, India; ^3^Department of Crop Physiology, University of Agricultural Sciences, GKVK, Bangalore, India; ^4^Department of Seed Science and Technology, University of Agricultural Sciences, GKVK, Bangalore, India; ^5^Plant Physiology, Matthias-Schleiden Institute, Friedrich-Schiller – University, Jena, Germany

**Keywords:** agriculture, abiotic stress, biotic stress, agrochemicals, crop breeding

## Abstract

Endophytes are non-disease causing microbes (bacteria and fungi) surviving in living tissues of plants. Their intimate association and possible coevolution with their plant partners have resulted in them contributing to an array of plant growth benefits ranging from enhanced growth and biomass accumulation, tolerance to abiotic and biotic stresses and in nutrient acquisition. The last couple of decades have witnessed a burgeoning literature on the role of endophytes (Class 3 type) in regulating plant growth and development and their adaptation to abiotic and biotic stresses. Though the underlying mechanisms of plant-endophyte interactions are far from clear, several studies have raised the hope of their potential application in agriculture, especially in mitigating abiotic and biotic stresses. The use of endophytes is envisaged as a route to reduce the production cost and burden on the environment by lessening the dependence on breeding for crop improvement and agrochemicals. Unfortunately, save a few well documented examples of their use, a little of these insights has been translated into actual agricultural applications. Here, we reflect on this paucity and elaborate on some of the important bottlenecks that might stand in way of fully realizing the potential that endophytes hold for crop improvement. We stress the need to study various facets of the endophyte-plant association for their gainful application in agriculture.

## Introduction

Endophytes are microbes residing within plants without causing any harm to their growth and development. Unlike disease-causing microorganisms, endophytes are non-pathogenic and many of them are known to enhance their plant host’s fitness (Mendes et al., 2013; Philippot et al., 2013). Fungal endophytes (FE) are classified in to four Classes based on their symbiotic criteria (Rodriguez et al., 2009). Class 1 endophytes are Clavicipitaceous fungi which survive in some cool season grasses and are transmitted vertically with their seeds. Class 2 endophytes colonize extensively the shoot, root, and rhizome of many plants and are transmitted both vertically and horizontally. Class 3 endophytes have a broad host range exhibiting restricted colonization of the shoot; they are transmitted horizontally. Class 4 endophytes which are also horizontally transmitted are restricted to the roots. The Class 3 endophytes which we address here, are effective in combating several abiotic stresses faced by their host plants, such as drought, salinity, nutrient deficiency, and metal toxicity, etc., and biotic stresses caused by pathogens and insect pests (Waller et al., 2005; Hardoim et al., 2008; Rho et al., 2018a; Manasa et al., 2020; Sampangi-Ramaiah et al., 2020). They are also known to produce pharmaceutically important secondary metabolites and enzymes (Shweta et al., 2010; Kusari et al., 2013; Kaushik et al., 2014; Kumara et al., 2014; Nagarajan et al., 2014; Uzma et al., 2019) and phyto-hormones (Bilal et al., 2017, 2018). In the past few decades, it became obvious that endophytes could be isolated from every plant studied (Strobel and Daisy, 2003; Hardoim et al., 2015; Suryanarayanan et al., 2018a; Giauque et al., 2019). These analyses showed that many attributes of endophytes, in particular their universal occurrence, sustained presence in plants, non-pathogenic nature, ability to enhance the biotic and abiotic stress tolerance of their plant hosts (Rodriguez et al., 2009), increase access to soil nutrients and increase the plant yield (White et al., 2019; Xia et al., 2019) project them as candidates holding high promise for use in crop improvement. Despite this, very few of the benefits associated with endophytes have been translated into real-world agricultural applications. Here, we reflect upon this gap and identify potential bottlenecks that might hinder the exploitation of endophytes in agricultural applications. We also discuss the possible approaches that might help pave the way ahead in allowing for a gainful application of endophytes in agriculture.

## Proof of the Principle of Application of Endophytes in Agriculture

Although fungal endophyte presence in plants is well known, the mechanism of plant colonization by these fungi is hardly known. For instance, the ability of species of *Colletotrichum*, *Guignardia*, *Pestalotiopsis*, *Diaporthe*, and *Xylaria* to infect a wide range of plant species as foliar endophyte (Suryanarayanan et al., 2018a) has not been explained. One study shows that an endophytic *Pestalotiopsis* produces a chitin deacetylase enzyme, which modifies its chitin cell wall to escape detection by its plant host immune system (Cord-Landwehr et al., 2016). Yuan et al. (2019), based on transcriptomics and proteomics analysis conclude that the endophyte *Gilmaniella* sp. infects its host plant *Atractylodes lancea* by reducing its immune response. A leaf is usually colonized by many Class 3 endophyte species (Rodriguez et al., 2009) exhibiting restricted growth in the tissue. Of these, invariably one or two species dominate the endophyte assemblage of the leaf, while the rest occur as satellite species with low colonization frequencies (Suryanarayanan et al., 2018a; Vaz et al., 2018). The interactions of a foliar endophyte with co-occurring endophytes (fungal and bacterial) in the leaf are little understood. It is possible that such interactions among them as well as their cross talk with the host would ultimately define the composition and ecological functions of the endophytes. According to Schulz et al. (2015), a balanced antagonism operates among the various endophytes in a plant tissue to maintain the endophyte community. For instance, an endophytic *Alternaria tenuissima* produced more antifungal polyketide stemphyperylenol in the presence of another endophyte, *Nigrospora sphaerica* (Chagas et al., 2013). Colonization by alien endophytes of a plant tissue is generally inhibited by the existing native endophytes (Mohandoss and Suryanarayanan, 2009; Suryanarayanan et al., 2018b).

Additionally, since the plant and its associated microbiome (which includes the endophytes) function as a mini ecosystem (the holobiome), to use endophytes gainfully it is essential to discern the different interactions operating here. Currently, there is very little information available on the functional aspect plant and its microbiome (Vandenkoornhuyse et al., 2015). We hardly know the roles of the core (dominant) and satellite (showing low degree of tissue colonization) endophyte species or of the ecological functions of key stone species in an endophyte assemblage. Plants generally resist infection by pathogens through pathogen-associated molecular pattern (PAMP)-triggered immunity (PTI) or effector-triggered immunity (ETI). The basic question of how the endophytes overcome such resistance responses while infecting the host has not been answered satisfactorily (Vandenkoornhuyse et al., 2015). This is intriguing since some symptomless pathogens infect plant tissues and survive as endophytes.

Despite such lacunae, work conducted over the last 2 decades across a range of agricultural crop plants has provided a strong proof of principle for the application of endophytes to agriculture (Suryanarayanan et al., 2017). The goal of this review is to highlight some of the salient studies to reiterate the potential application of endophytes in agriculture but not to review the literature on plant/endophyte interactions.

Laboratory experiments and glasshouse trials strongly indicate that endophytes could mitigate stresses in agriculturally important crops and increase productivity. A recent meta-analysis by Rho et al. (2018a) highlights the potential applications of endophytes in mitigating drought, salinity, and nutrient shortfalls in agricultural systems. For instance, inoculation of FE from wild barley in to a barley cultivar significantly increased its grain yield (Murphy et al., 2018). Treatment of wheat plants with the endophyte *Alternaria alternata* enhanced growth and imparted drought tolerance. Plants colonized by the endophyte effectively quenched stress-induced free radicals and also accumulated higher levels of osmolytes (Qiang et al., 2019). Growth promotion induced by endophyte could often be brought about indirectly as evident in peanut plants where the endophyte, *Phomopsis liquidambri* enhanced nodulation and nitrogen fixation by H_2_O_2_ and NO signaling (Xie et al., 2017). FE protect crops against abiotic stresses under laboratory conditions, as shown for salt (Baltruschat et al., 2008; Manasa et al., 2020; Sampangi-Ramaiah et al., 2020), heat and drought (Redman et al., 2002; Bailey et al., 2006; Hubbard et al., 2014; Ali et al., 2018; Sangamesh et al., 2018) stresses. A number of studies confirm that the root endophyte *Piriformospora indica* (*Serendipita indica*) ameliorates a broad range of abiotic stresses in many crop plants. In *Zea mays*, it solubilizes the insoluble phosphate in the soil, which is unavailable to the plant and transports it to the plant (Yadav et al., 2010; Aslam et al., 2019), increases the drought stress tolerance of barley (Ghaffari et al., 2019), and, apart from improving stress tolerance, increases growth and nutrient acquisition in soybean plants (Bajaj et al., 2018). Endophytes also enhance tolerance of host plants to biotic stressors including pathogenic fungi (*Botrytis cinerea* in grapevine – Barka et al., 2002; *Phytophthora* sp. in cocoa – Arnold et al., 2003; *Cronartium ribicola* in *Pinus monticola* – Ganley et al., 2008; *Verticillium dahliae* in tomato – Fakhro et al., 2010, and *Phytophthora capsici* in hot pepper – Bae et al., 2011). The protection of plants against insect pests is well documented for Class 1 FE (Rodriguez et al., 2009), which are vertically transmitted within their grass host communities (Rodriguez et al., 2009; Kauppinen et al., 2016; Raman and Suryanarayanan, 2017), although, according to Faeth and Fagan (2002), such endophytes may not function as defensive mutualists in native plants. A few horizontally transmitted Class 3 FE are also reported to reduce insect attack of plants (Vega, 2008; Vidal and Jaber, 2015). The action of endophytes in plants may often be due to the synergy with other co-existing endophytes, fungi, or bacteria as was demonstrated by Bilal et al. (2020). They showed that the two EF, *Paecilomyces formosus* and *Pencillium funiculosum* acted synergistically to impart tolerance to drought, high temperature, and heavy metals (Bilal et al., 2020).

The underlying mechanisms of the benefits of endophytes on their host plants are currently being unraveled. It appears that a combination of alterations in the gene expression and physiology of the host induced by the endophyte is reflected as the plant’s response to stresses. But much of the studies investigating the mechanisms are largely restricted to a few fungi, notably the root endophyte, *Pirimiforma indica*. For example, in rice, *P. indica* confers drought tolerance by regulating miR159/miR396 that target MYB and GRF transcription factors, involved in regulation of growth and hyposensitivity response (Mohseni Fard et al., 2017). In yet another study in rice, *P. indica* colonization led to the differential miRNA synthesis that targeted transcription factors involved in nutrient uptake, Na^+^ transport, and growth regulation including auxin responsive proteins (Kord et al., 2019). In soybean, *P. indica* colonization leads to the upregulation of genes within the phenylpropanoid and derivative pathway and in iron scavenging siderophores (Bajaj et al., 2018). More recently, a comparative transcriptome analysis of rice colonized by a salt adapted EF was shown to upregulate a number of genes involved in both abiotic and biotic stress tolerance, when the plants were subjected to salinity stress (Sampangi-Ramaiah et al., 2020). There is now increasing evidence that the endophyte effects on plants are mediated through specific signaling cascades which, upon perception by the host cell, alter host gene expression (Sampangi-Ramaiah et al., 2020).

Infection of a plant by FE rapidly upregulates defense related genes and the lignin and cellulose content of its cell walls (Soliman et al., 2013; Mejía et al., 2014); such responses of the plant as well as the chemicals of the resident endophyte enhance its ability to counter abiotic and biotic stressors (Estrada et al., 2013). Thus, it is apparent that the use of endophytes is a promising route for improving crop productivity by reducing the dependence on breeding and agrochemicals. However, the cost suffered by the plant for harboring endophytes is a facet that has not been understood adequately. For the plant host, the presence of FE results in reduced photosynthesis, altered host nitrogen metabolism, and loss of photosynthates (Mejía et al., 2014). Alternatively, the hypothesis that respiratory CO_2_ of endophytes could result in islands of low photorespiration thus enhancing photosynthesis in the leaf tissue (Suryanarayanan, 2013) appears to be true at least with reference to bacterial endophytes (Rho et al., 2018b). To fully appreciate the benefit accrued by endophyte association, the cost-benefit ratio for a plant should ideally be worked out by taking into account the entire community of endophyte it harbors.

In summary there is ample evidence to suggest that endophytes can mediate growth and other benefits such as adaptation to abiotic and biotic stresses in plants that could in principle lead to their gainful application in agriculture.

## From the Lab to the Field: Still a Chasm

Long term studies confirm that plant association with mycorrhizal fungi is not accidental and results in increased stress tolerance of the associated plant (Gehring et al., 2017). It is conceivable that the adaptive capabilities of the host plant increase substantially due to the extensive metabolic potential of the associated mycorrhizal fungi (Lau et al., 2017). Such focused studies on endophyte association are lacking though endophyte technology has distinct advantage over inorganic agriculture practices. Since most of the endophytes used in agriculture colonize the underground and above ground tissues and develop together with their host plants, their metabolisms are adapted to each other. The balanced interactions during the entire symbiotic phase allows for better adaptation to environmental changes since the responses are the result of a synergistic interaction between the two partners, which is believed to be more than the sum of the responses of the two partners alone (Rosenberg and Zilber-Rosenberg, 2018). However, the successful use of these microbes depends largely on their performance under field conditions which now requires extensive research addressing the barriers for effective product development.

There are many publications endorsing the positive role of FE on plant growth and performance in adverse environments (Gundel et al., 2013). *Epichloë* (Class 1) endophyte strains selected for low toxicity to livestock and which increase the productivity of forage grasses and the robustness of turf grasses have been used in the United States, Australia, and New Zealand (Young et al., 2014; Kauppinen et al., 2016). The performance of *Epichloë* endophytes infected grasses is superior such that Kauppinen et al. (2016) assert their use while designing sustainable management strategies for agriculture. According to Johnson et al. (2013) endophyte mediated plant trait improvement contributed around $200 million per annum to the economy of New Zealand. Root endophytic *Trichoderma* species increase yields of stressed crops by inducing biochemical pathways, which render the toxic reactive oxygen species generated during stress into less toxic compounds (Harman et al., 2020). Despite such studies, with reference to non-*Epichloë* endophytes, there is very little by way of commercial products which are evident in the global market. To evaluate this, we searched the United States and Indian patent database to analyze patents filed with respect to endophyte treatment for plant health benefits. The comprehensive search was made using the keywords “endophyte” and “plant” and the exclusive patents typically describing the effective utilization of endophytes for plant benefits are compiled in Table 1.^1^

**Table 1:** United States patents claiming endophyte’s benefit in plants (2000–2018).

No	Organism	Patent claim	Patent #	Patent author
**A. Endophytic fungi**				
1	*Neotyphodium*	Resistance to invertebrate pests	61,11,170	Latch et al., 2000
2	Fungi	Insect resistance, disease resistance, drought resistance	61,80,855	Hiruma, 2001
3	*Neotyphodium* spp. or *Gliocladium* spp.	Insect resistance, disease resistance to Italian rye grass	65,48,745	Hiruma et al., 2003
4	*Neotyphodium* spp.	Pest control, no toxicity to grazing animal	68,05,859	Imada et al., 2004
5	*Muscodor albus* and *Muscodor roseus*	Pest control by volatiles	69,11,338	Strobel et al., 2005
6	*Muscodor albus*	Bio control of fungus	77,54,203	Strobel et al., 2010
7	*Neotyphodium lolii*	No ryegrass toxicosis, enhance growth under drought	79,76,857	Tapper et al., 2011
8	*Colletotrichum dematium*	Antifungal peptide	80,80,256	Strobel et al., 2011
9	*Muscodor albus* and *Muscodor roseus*	Disease and nematode resistance by volatile	80,93,024	Strobel et al., 2012
10	*Colletotrichum dematium*	Biological activity against either Botrytis cinerea, Sclerotinia sclerotiorum, or Rhizoctonia solani	87,65,147	Strobel et al., 2014
11	*Acremonium* spp.	Protection of grass plants from biotic or abiotic stress	89,75,489	Carven, 2015
12	*Clonostachys rosea*	Stimulation of nodules in legumes, enhanced plant growth under stress	81,01,551	Stewart et al., 2012
13	*Dothideomycetes* spp.	Increased boll retention, growth, and yield. Resistance to drought, cold, metal, salt, fungi, bacteria, virus and pests in cotton	92,77,751	Sword, 2016
14	*Neotyphodium lolii*	No ryegrass toxicosis, protection from pests and/or abiotic stress	93,74,973	Tapper et al., 2016
15	*Lophodermium* spp.	Antifungal activity in pine	94,69,836	Miller et al., 2016
16	*Phialocephala* spp.	Pest tolerance in conifers	95,49,528	Miller et al., 2017
17	*Sarocladium* spp.	Promotion of germination, resistance to nitrogen stress	96,87,001	Vujanovic et al., 2017
18	*Neotyphodium coenophialum*	No ergopeptide, black beetle resistance, enhanced biomass and yield in grasses	97,06,779	Roulund et al., 2017
19	Incertaesedis, Nectriaceae, or Plectosphaerellaceae	Improved tolerance to drought, pests, better yield in cotton	97,56,865	Sword, 2017
20	*Acremonium* spp.	Improved resistance to diseases and/or pests in Brachiariaurochloa grass	98,72,502	Spangenberg et al., 2018
21	*Trichoderma harzianum*	Enhanced growth or seed germination under abiotic stress	99,61,904	Rodriguez et al., 2018

We observed that a considerable number of fungal endophytes were acclaimed to improve the overall agronomic attributes; sometimes, an endophyte confers more than one beneficial effect on plant growth and yield. For example, in one case endophytes not only improved drought tolerance, but also reduced pest attack, and improved the yield attributes in cotton (United States patent # 9,277,751; Table 1). Many FE control pests and diseases in plants (Table 1). Only four patents are listed in the Indian database,^2^ of which only that of Arora et al. (2015) has been experimentally examined and approved (Table 2).

**Table 2:** Indian patents claiming endophyte’s benefit in plants (2000–2018).

No	Organism	Patent claim	Patent #	Patent author
**A. Endophytic fungi**				
1	*Piriformospora indica* and *Azotobacter chroococcum*	Plant growth promotion	2017/DEL/2013	Arora et al., 2015
**B. Endophytic fungi + Bacteria**				
2	Fungi and bacteria	Patent awaiting improvement of germination rate, emergence rate, shoot biomass, root biomass, seedling root length, seedling shoot length, and yield.	201717043115	Karen et al., 2017
3	Fungi and bacteria	Patent awaiting improvement of agronomically important traits.	201717043114	Karen et al., 2018

We contacted 66 authors referred to in the meta-analysis publication of Rho et al. (2018a) and obtained responses from eight scientists, of which one claimed translation of the research into a commercial product (Cheng et al., 2012). This demonstrates that product development is disappointingly low compared to the scientific investments. As mentioned by one respondent, the complex and protracted regulatory guidelines are mainly responsible for the low success rate in commercialization of the product developed by the scientific community.

Finally, we also compiled information on commercial endophytic products, which are already available in the market. An example is endophyte infected grasses commercialized by a New Zealand based company “Grasslanz”.^3^ The inoculation of an endophyte in the grass grown in airports and recreational areas deter insects, grazing animals, and birds. The “Grasslanz” homepage claims over 69 and 88% reduction in above and below ground insect pests, respectively. This application might be a long-term solution for bird strike issues in the airports.^4^ Another example comes from “BioEnsure” developed by Adaptive Symbiotic Technologies (Seattle, United States). “BioEnsure” is endophytic fungal based preparation which had shown between 3 and 30% increase in crop (corn, rice, wheat, soybean, and cotton) yield under drought, heat, and cold stresses.^5^

## Bottlenecks and Way Forward

**Figure 1:**
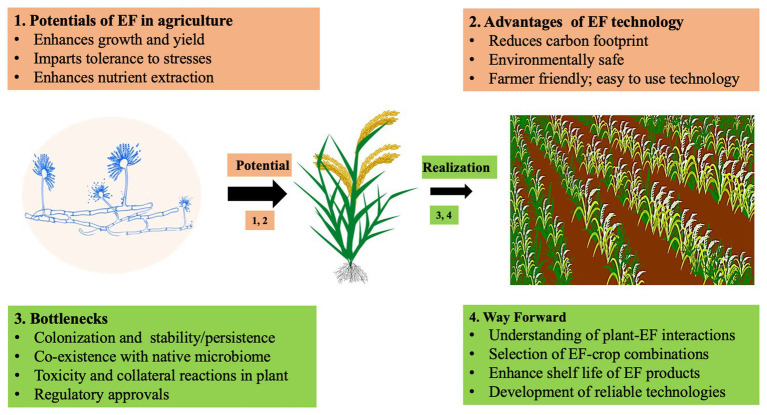
Schematic illustration of the potentials and bottlenecks of application of endophytes in agriculture. Please see text for explanation.

Taking FE as a standard, we discuss the constraints faced in using endophytes for crop improvement (Figure 1). The two basic steps for successful use of FE in crop improvement are: (1) the identification of a FE with a needed trait and (2) introduction of that FE into the crop which involves colonization, establishment, and confirmation of a successful symbiosis and sustained expression of the desired trait in FE-colonized crops (Hyde et al., 2019). The first step is straight forward and involves screening of FE isolated from plants for choice traits. The second step is fraught with uncertainties since it entails the interaction of the newly introduced FE with the crop host and its already existing endobiome. Here, the vertically transmitted grass endophyte *Epichloë* appears to be more tractable owing to its tight host specificity and systemic and sustained infection of the host (Saikkonen et al., 2016) though certain questions like the role of genetics in dictating the fungus-grass symbiosis and its effect on natural and agricultural grass ecosystems remain unanswered (Saikkonen et al., 2016). Relatively less information is available on the interactions of horizontally transmitted endophytes with their hosts. Although the broad host range of some of these like *Colletotrichum*, *Guignardia* (*Phyllosticta*), *Pestalotiopsis*, *Diaporthe* (*Phomopsis*), and *Xylaria* could be a salient feature in ensuring successful colonization of different crops, their influence on the native microbes of the host crop and higher trophic levels are not known. (Rabiey et al., 2019). Furthermore, many species of these FE genera are latent pathogens and it is possible that climate change could tilt their lifestyle toward pathogenicity (Moricca and Ragazzi, 2008; Paolinelli-Alfonso et al., 2016). These facets of FE interactions should be unraveled for their efficient use in agriculture. Suffice it to say that little information with agricultural relevance is available for this step. Cogitation on the plant’s defense reactions toward biotrophic and necrotophic fungal pathogens at the structural, biochemical, and genome levels could be of heuristic value here.

First, as many fungi including endophytes produce toxic secondary metabolites including mycotoxins (Thirumalai et al., 2013, 2020), it is necessary to evaluate endophytes for the production of such metabolites when they are introduced in to crop plants; this becomes even more important if the endophytes reach the edible parts of the plant, such as the seeds or tuber (for human consumption) or forage (for animal consumption; Figure 1). Second, there is little information on the interplay that operates between the newly introduced microbes and the native plant endobiome. Hardly any information is available on the infection of a plant host by an endophyte, which is the very first step of FE establishment. One explanation for the colonization of a wide range of plants by non-host specific FE is that they escape detection by the plant immune response by altering the chitin in their cell wall, while infecting the plant (Cord-Landwehr et al., 2016). Endophyte enrichment technology should ensure that the introduced endophyte establishes itself in the plant host endobiome and its introduction does not perturb the native microbiome, which could result in negative impacts on plant performance or the environment/ecosystem or agrosystem (Figure 1). For instance, the presence of non-native endophytes which have not coevolved with the host could eliminate the native beneficial microbes resulting in a net loss for the plant (Whipps, 2001). Galls in trees induced by wasps are abscised by necrosis induced by the fungal endophyte *Apiognomonia errabunda*; however, this is more harmful to the host than the galls (Sieber, 2007). Thus, it is essential to consider the plant as a holobiont, i.e., the plant together with the diversity of microbes interacting with the plant and each other to achieve FE mediated enhance plant performance (Rosenberg and Zilber-Rosenberg, 2018; Rabiey et al., 2019).

The plant and its microbiome have to be considered as a mini-ecosystem in which the microbiome can be the essential determinant for plant performance (Agler et al., 2016). For example, Thynne et al. (2019) showed that fungal pathogens harboring horizontally acquired modular polyketide genes from bacteria exhibit a broader host range than those that are not housing these trans-genes. This highlights the importance of short-term and long-term interactions between plant and microbes and might have an important influence on the survival of a newly introduced endophyte. Furthermore, in the halophyte *Salicornia europaea*, the fungal endophyte community is influenced by the bacterial community but not vice versa (Furtado et al., 2019). Endophytes which do not produce antibiotics in pure cultures do so when co-cultured with another endophyte species (Schulz et al., 2015) suggesting that microbial interactions can profoundly influence the endobiome. It is conceivable that the complex network of such interactions (Schulz et al., 2015) can result in warding off the introduced non-native endophyte in the crop, or that the newly introduced endophyte alters the endobiome community such that it is no longer beneficial for the plant. The report that mango leaves could be colonized by alien FE species only after the native EF species are eliminated by systemic fungicide treatment is an example for the importance for this scenario (Mohandoss and Suryanarayanan, 2009). Similarly, an FE from a brown seaweed which effectively controls the insect pest *Helicoverpa armigera* in crops does not survive in the new host, even after infection with high spore doses, probably due to its poor competitive ability with the native microbiome (Suryanarayanan et al., 2018b). Alternatively, it is reasonable to assume that a new endophyte can dominate the holobiome and restrict growth of the endogenous microbes, which can result in loss of benefits for the plants. In this context, it is important to mention that barely anything is known about the role of newly introduced endophytes on existing mycorrhiza in crop plants, although many examples demonstrate that both symbionts can co-exist in the same root. Yet another factor to be considered is the possible negative effect of an introduced endophyte which could nullify the beneficial effect provided by it (Rabiey et al., 2019). These include lack of substantial information on (1) field performance of endophytes, (2) influence of weather conditions on endophyte performance, (3) highly restricted colonization of plants leading to only localized results, and (4) alteration in the native community of associated microbes by the endophyte leading to reduced performance of the hoist (Rabiey et al., 2019).

Third, it is necessary to lay caution on the possibility that an introduced endophyte may elicit disease reactions in a non-host plant. Fungi such as *Leptosphaerulina crassiasca* (Suryanarayanan and Murali, 2006) and *Fusarium verticillioides* (Oren et al., 2003) could live in a plant as endophytes or pathogens. Endophytes alter their lifestyle depending on host genotype, web of interactions, the experience with co-occurring microbes in the endobiome, and the environmental conditions (Hardoim et al., 2015). Symptomless FE could become pathogenic due to heat stress such as that produced by climate change (Paolinelli-Alfonso et al., 2016). Factors which determine the lifestyle of a microbe in its host environment and influence its expression profile need to be understood to effectively use FE in crop management (Figure 1). Such investigations can be done in three directions – by analyzing the influences of the host milieu, of the microbes with and without the host, and of environmental conditions in which the crop is growing. Furthermore, for FE to be important for agricultural applications, it should be ensured that endophyte formulations maintain substantial viability and activity (Woodward et al., 2012). They also have to be consistent with cultural practices since agrochemicals can have a strong influence on EF communities associated with crops (Stuart et al., 2018).

Finally, crop breeding programs have not considered beneficial microbiota yet (Bakker et al., 2012; Raaijmakers and Mazzola, 2016), and genetic markers favoring endophyte association with crops are not known. Plant genetic loci controlling endophyte colonization in crop plants might be a helpful tool to promote agriculture with less agrochemical usage. Since information on the influence of agricultural practices on FE of crops is meager, the performance of FE in real field situations cannot be predicted. A recent study reports that pest and pathogen management alters the leaf microbiome diversity of tea (Cernava et al., 2019). Agricultural practices including fertilizer application and mowing frequency influence the FE communities in agriculturally important grasses (Wemheuer et al., 2019). Application of agrochemicals such as triflumuron and fenoxaprop-P-ethyl decreases the diversity, richness, and evenness of FE in soybean (da Costa Stuart et al., 2018). Gange et al. (2019), conclude that although endophytes could be crucial players in plant protection, experimental methodology, and inoculation methods to reintroduce FE into plant can skew the results. Since environment and agricultural practices influence interactions within the endobiome, these factors have to be taken into consideration for using FE in crop improvement (Figure 1). Since plant genotype influences the diversity and composition of its microbiome (Li et al., 2018), an FE effective for one crop may not be suitable for other crops. Hence, developing crop varieties which would readily accommodate an introduced FE will improve crop production and productivity (Schlatter et al., 2017).

Another formidable challenge for many biofertilizers, biopesticides, or bio-stimulants used in agriculture is obtaining registration for the product. The licensing laws differ with countries and registration may involve several regulatory bodies which is time-consuming (Timmusk et al., 2017). Therefore, this non-biological bottleneck requires more efficient administration such that research on endophyte-mediated crop improvement could be galvanized (Harman et al., 2010).

## Conclusion

From the disparate studies so far, it seems that a deep understanding of the holobiont ecology and breeding of crops for better association with beneficial microbes (particularly endophytes) is the ideal way forward. Commercialization of endophytes and/or their products can promote plant fitness and agricultural yields. In spite of an increasing demand for these biological products in today’s market, we still need to address various gridlocks related to basic mechanisms of newly introduced FE into plants and eco-agrosystems. Field applications will help to understand whether results from the laboratory reflect those from the real world, in particular under adverse climate conditions.

## Author Contributions

RS along with VC planned the review. VC collated the data and prepared the draft. TS contributed to redrafting the manuscript and adding substantially to the section on the bottlenecks and way-forward and straightening the bibliography. KN, SP, and RO edited and contributed to several rounds of revision of the manuscript. All authors contributed to the article and approved the submitted version.

### Conflict of Interest

The authors declare that the research was conducted in the absence of any commercial or financial relationships that could be construed as a potential conflict of interest.

## Footnotes

^1^http://patft.uspto.gov/netahtml/PTO/search-bool.html; accessed on March 19, 2019

^2^http://ipindiaservices.gov.in/publicsearch; accessed on March 20, 2019

^3^http://www.grasslanz.com/

^4^http://www.grasslanz.com/understanding-the-science

^5^cf. http://www.adaptivesymbiotictechnologies.com/field-results.html
